# Incidence of Influenza in Healthy Adults and Healthcare Workers: A Systematic Review and Meta-Analysis

**DOI:** 10.1371/journal.pone.0026239

**Published:** 2011-10-18

**Authors:** Stefan P. Kuster, Prakesh S. Shah, Brenda L. Coleman, Po-Po Lam, Agnes Tong, Anne Wormsbecker, Allison McGeer

**Affiliations:** 1 Mount Sinai Hospital, Toronto Ontario, Canada; 2 University of Toronto, Toronto Ontario, Canada; 3 Ontario Agency for Health Protection and Promotion, Toronto Ontario, Canada; 4 Hospital for Sick Children, Toronto Ontario, Canada; Centers for Disease Control and Prevention, United States of America

## Abstract

**Background:**

Working in healthcare is often considered a risk factor for influenza; however, this risk has not been quantified. We aimed to systematically review evidence describing the annual incidence of influenza among healthy adults and healthcare workers (HCWs).

**Methods and Findings:**

We searched OVID MEDLINE (1950 to 2010), EMBASE (1947 to 2010) and reference lists of identified articles. Observational studies or randomized trials reporting full season or annual influenza infection rates for healthy, working age adult subjects and HCWs were included. Influenza infection was defined as a four-fold rise in antibody titer, or positive viral culture or polymerase chain reaction.

From 24,707 citations, 29 studies covering 97 influenza seasons with 58,245 study participants were included. Pooled influenza incidence rates (IR) (95% confidence intervals (CI)) per 100 HCWs per season and corresponding incidence rate ratios (IRR) (95% CI) as compared to healthy adults were as follows. All infections: IR 18.7 (95% CI, 15.8 to 22.1), IRR 3.4 (95% CI, 1.2 to 5.7) in unvaccinated HCWs; IR 6.5 (95% CI, 4.6 to 9.1), IRR 5.4 (95% CI, 2.8 to 8.0) in vaccinated HCWs. Symptomatic infections: IR 7.5 (95% CI, 4.9 to 11.7), IRR 1.5 (95% CI, 0.4 to 2.5) in unvaccinated HCWs, IR 4.8 (95% CI, 3.2 to 7.2), IRR 1.6 (95% CI, 0.5 to 2.7) in vaccinated HCWs.

**Conclusions:**

Compared to adults working in non-healthcare settings, HCWs are at significantly higher risk of influenza.

## Introduction

One frequently postulated risk factor for influenza infection is being a healthcare provider [1]. Outbreaks of influenza in long term care facilities are very common, occurring in as many as 50% of facilities each year [2], and there have been numerous reports of outbreaks of influenza in acute care hospitals [3]. The assumption of transmission of influenza from patients to healthcare workers (HCWs) and vice versa seems obvious, as influenza is transmitted primarily by close contact, and many HCWs have close contact with persons ill due to influenza. On the other hand, influenza is also very common in the community mainly because children are most often affected, and household transmission of influenza is frequent [4]–[8]. Only one study during a single season has prospectively assessed the risk of serologically proven influenza infection associated with HCW status in a large, multicenter cohort. Although no association was found, the upper bound of the confidence interval of the point estimate did not exclude a potentially large increase in risk [9]. Studies of influenza risk comparing HCWs to other adults are challenging in that adequate sample sizes are difficult to achieve, and other risk factors for adults are not well described. However, many studies have reported rates of influenza in working adults, and others have reported rates among HCWs. We aimed to systematically review evidence describing the annual incidence of influenza among healthy adults and HCWs, and to evaluate the hypothesis that influenza incidence rates are higher in HCWs than in other healthy, community-dwelling adults.

## Materials and Methods

We follow the meta-analyses of observational studies in epidemiology (MOOSE) guidelines for reporting our results [10]. Because of relative lack of direct comparative studies between HCWs and non-HCW adults, we extrapolated information regarding risk of influenza among both populations from individual studies and performed meta-analyses of rates.

### Search strategy

We identified all relevant studies in the English language literature, searching OVID MEDLINE (from 1950 to September 2010) and EMBASE (from 1947 to September 2010) with the help of an experienced librarian (detailed search strategy provided in Data S1). We also searched reference lists of included studies. We did not include conference proceedings, abstracts, theses, dissertations or national or local vital statistics data not published as peer reviewed articles.

### Study selection

#### Inclusion criteria

Observational studies (cohort and case-control studies) or randomized trials (studies of vaccines where data from each arm were analyzed separately for vaccinated and unvaccinated participants) that reported on healthy, working age adult subjects (as per the study definition, or persons 18 to 64 years of age) or HCWs with asymptomatic or symptomatic influenza infection who were assessed prospectively over one or more complete influenza seasons were included.

Studies must also have measured influenza infection by at least one of the following methods: (a) a four-fold rise in antibody titer comparing serum drawn pre- and post-season; (b) a four-fold rise in antibody titer comparing acute and convalescent serum obtained systematically from participants with acute respiratory/influenza-like illness; (c) culture or (d) PCR of nasopharyngeal aspirates or swabs obtained systematically from participants with acute respiratory/influenza-like illness.

#### Exclusion criteria

Case reports and case series where the denominator population could not be determined, studies that reported incidence rates in military personnel, college or university students, or persons in remote or isolated communities; outbreak reports; studies in which data for adults 18 to 64 years of age could not be separated from data for older adults and/or children (unless children accounted for <2% or older adults <10% of the study population, according to our a priori definition), studies with a duration of less than one complete influenza season, and studies of a single influenza strain or type only (unless the studied strain was reported to account for >90% of all circulating strains in the seasons studied) were excluded.

#### Selection

One review author (SPK) inspected the abstract of each reference identified by the search and selected the studies for full review. All possibly relevant articles were then inspected for inclusion in full by two review authors (SPK and AM). Discrepancy was resolved by consensus.

### Data extraction

Data from included studies were independently extracted by two reviewers; SPK (all studies) and one of the other reviewers (AM, BC, AW or PL), using a standardized data collection form. A third reviewer was consulted in case of disagreement between the two data extractors and discrepancy was resolved by consensus.

Data from included studies on year of publication, influenza seasons (years) under study, circulating influenza subtypes, study design, population (HCWs vs. adults working in non-healthcare settings vs. adults living with children in their households), country of origin, vaccination status of participants, diagnostic methods, number of subjects studied and number infected based on the outcome measures of interest (see below) were collected. When available from the reported results in the studies, data specifically for subjects 18 to 64 years of age were extracted.

### Outcome

The outcome measures of interest were the incidence rate of symptomatic infection and that of all infection (symptomatic and asymptomatic). Symptomatic infection was defined as acute illness consistent with influenza (as defined in each study), together with laboratory evidence for influenza (PCR or culture yielding influenza virus, or a four-fold or greater rise in antibody titer). All influenza infection was defined as a four-fold or greater rise in antibody titer over the influenza season, with or without other diagnostic tests, regardless of clinical symptoms.

### Assessment of risk of bias

Risk of bias among included studies was assessed in the domains of patient selection, outcome assessment and attrition by SPK (adapted from Newcastle-Ottawa Scale [11], [12], assessment of quality of included studies tool provided in Table S1). Overall risk of bias was assessed by selecting the greatest risk of bias among these three domains. Existing literature suggested that our exposures of interest (HCW status versus other adults working in non-healthcare settings) only sporadically matched that of published studies. Rather, other exposures, such as vaccination status, were commonly assessed in subpopulations that corresponded to our exposures of interest. Therefore, the full Newcastle-Ottawa scale [12], which mainly focuses on quality of exposure and outcomes, did not comply with the requirements for this study question.

### Data synthesis and analysis

*A priori*, we planned a stratified meta-analysis because of anticipated clinical heterogeneity among included studies. Subgroup categories were: symptomatic versus all infections; vaccinated versus unvaccinated participants; and studies of adults in households with children versus other studies of adults; and, within the subgroup of symptomatic infections: influenza symptoms with acute/convalescent or pre-/post-season serology versus PCR with or without viral culture versus viral culture alone.

Strata with at least two eligible studies were synthesized by conducting a meta-analysis of incidence rates. Variances around estimates of incidence rates from various studies were calculated. Binomial confidence intervals were calculated using SAS (version 9·1, SAS Institute, Cary, NC). Appropriate subgroups were meta-analyzed using inverse of variance for calculating weight for each estimate in the meta-analysis. The meta-analysis was performed using Review Manager software (RevMan version 5·0. Copenhagen: The Nordic Cochrane Centre, The Cochrane Collaboration, 2008). Because we anticipated heterogeneity between studies, a random-effects model was used for all analyses. Statistical heterogeneity was initially inspected graphically (forest plot) and assessed by calculating tests of heterogeneity using the Cochran Q test (Chi-square test). We quantified the degree of heterogeneity using the I^2^ statistic [13]. We also calculated incidence rate ratio (with 95% confidence interval [CI]) between vaccinated and unvaccinated adults and HCWs for the outcomes of all infections and symptomatic infections, respectively, using the delta method [14].

In secondary analysis, we asked whether differences in incidence could be detected between seasons in which different influenza subtypes (A/H1N1, A/H3N2 or B) predominated, and, for symptomatic infections, whether the incidence was different in studies with a requirement for fever (temperature ≥37.8°C) in the definition of influenza-like-illness.

## Results

A total of 7,763 (OVID MEDLINE) and 16,944 (EMBASE) titles and abstracts were screened, and 124 full articles were retrieved. Of these, 29 studies satisfied eligibility criteria and were included in the meta-analysis [5], [9], [15]–[41]. Characteristics of included studies are presented in Tables S2 and S3. Excluded studies and reasons for exclusion are listed in Data S2. Overall risk of bias was minimal in two studies [37], [39], low in 17 studies [5], [15], [16], [19]–[25], [29]–[31], [33], [38], [40], [41], moderate in eight studies [9], [17], [26], [27], [32], [34]–[36], and high in two studies (Table S4) [18], [28].

The included studies had data from a total of 97 influenza seasons between 1957 and 2009 with a total of 58,245 participants (18,131 subjects for all infections, and 40,114 subjects for symptomatic infections, respectively). Nine studies (38 influenza seasons, 13,373 participants) assessed influenza rates in HCWs [9], [19], [20], [27]–[29], [36], [40], [41], seven studies (20 influenza seasons, 3,642 participants) enrolled families with children [5], [21]–[24], [26], [31], and 14 studies (39 influenza seasons, 41,230 participants) assessed influenza rates in adults not working in healthcare settings and irrespective of family status [15]–[18], [20], [25], [30], [32]–[35], [37]–[39]. From these, one study provided data for both HCWs and non-HCWs without comparison [20]. One other study compared HCWs to non-HCWs directly, but the non-HCW group did not fit our inclusion and exclusion criteria as college students were included in the non-HCW group [9].

**Figure 1:**
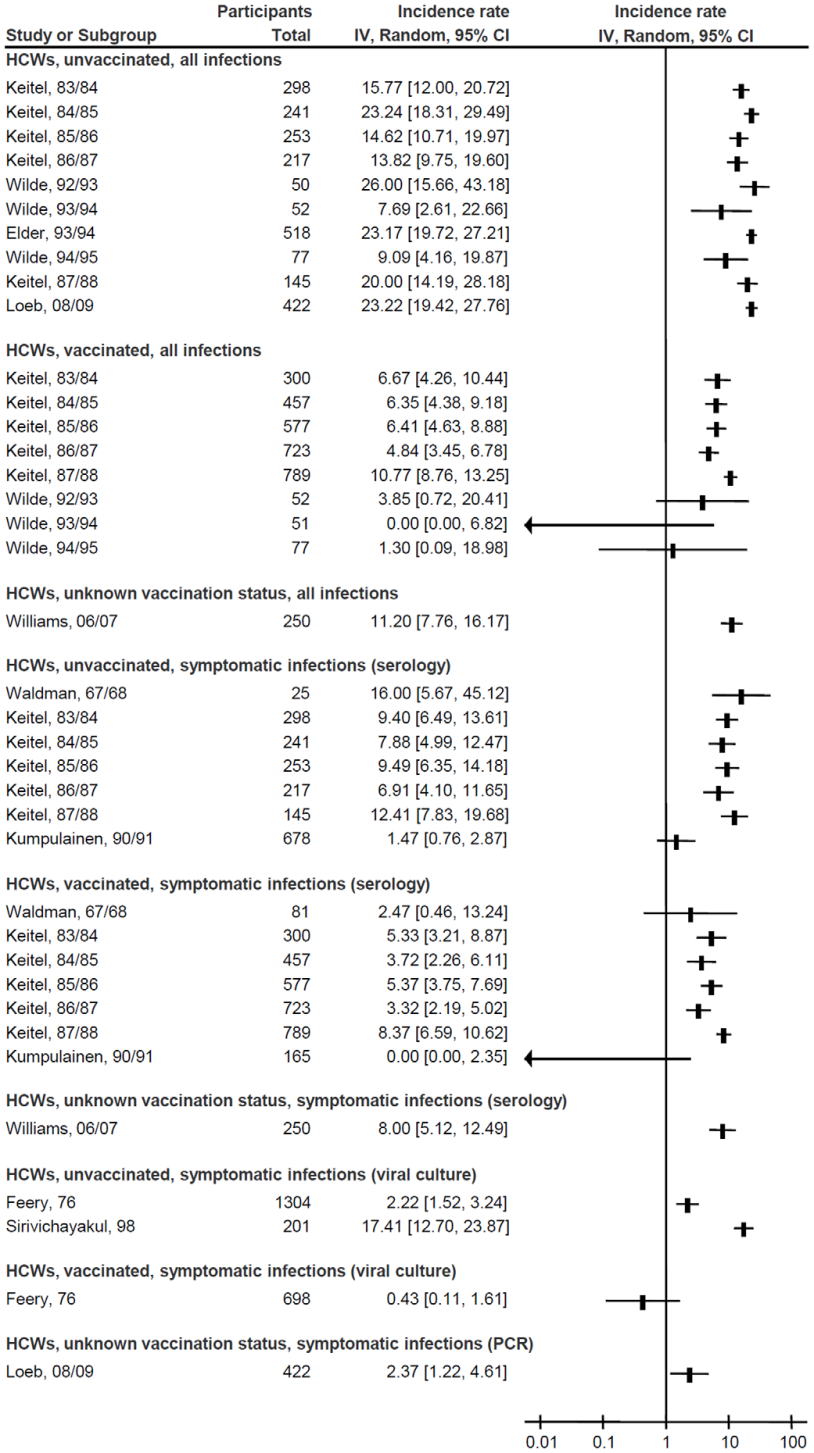
Forest plot of influenza incidence rates in healthcare workers. Squares and horizontal lines through the squares represent incidence rates with 95% confidence intervals. Abbreviations: IV, inverse variance; CI, confidence interval; HCWs, healthcare workers; PCR, polymerase chain reaction.

**Figure 2:**
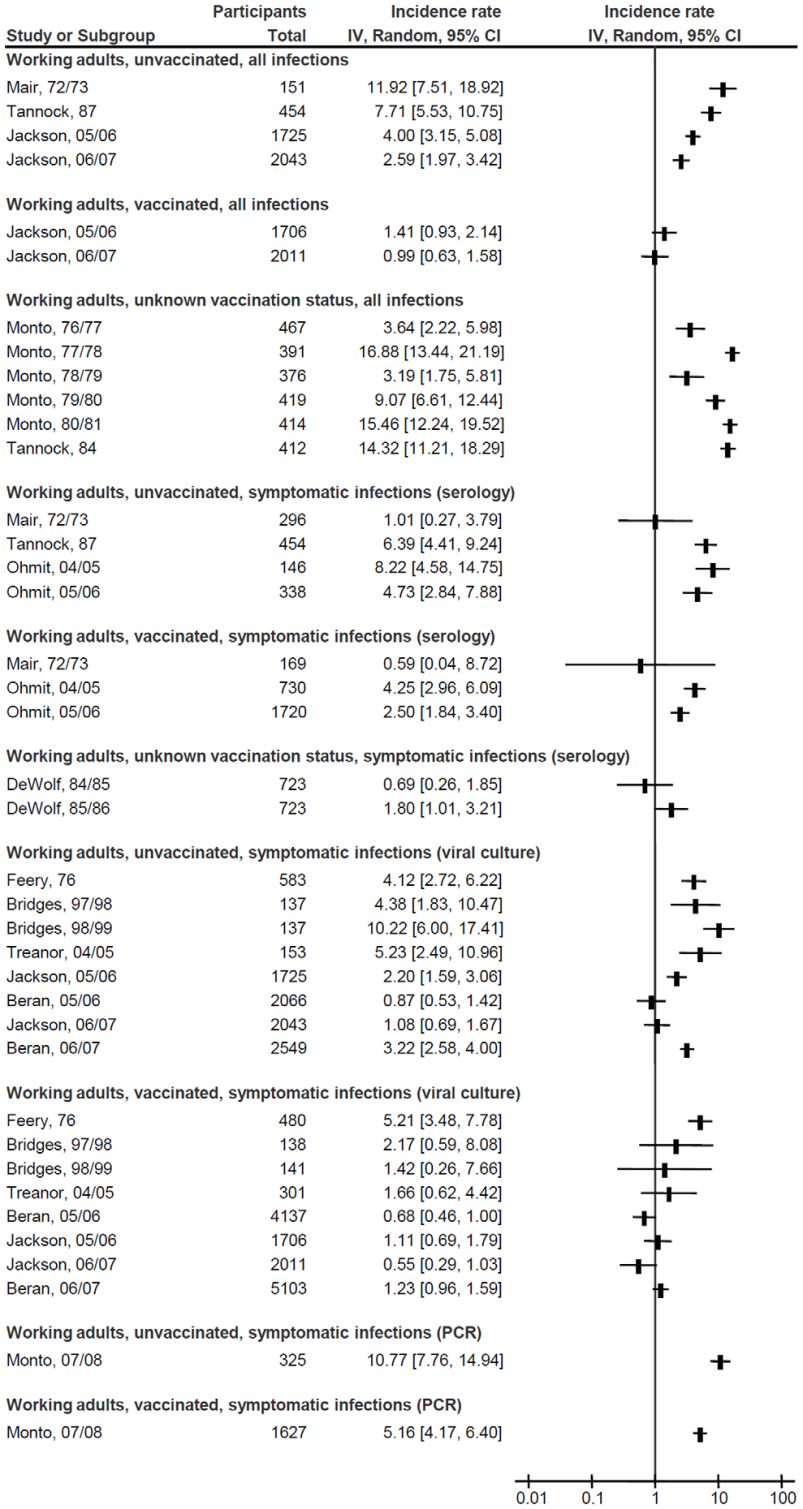
Forest plot of influenza incidence rates in adults working in non-healthcare settings. Squares and horizontal lines through the squares represent incidence rates with 95% confidence intervals. Abbreviations: IV, inverse variance; CI, confidence interval; PCR, polymerase chain reaction.

**Figure 3:**
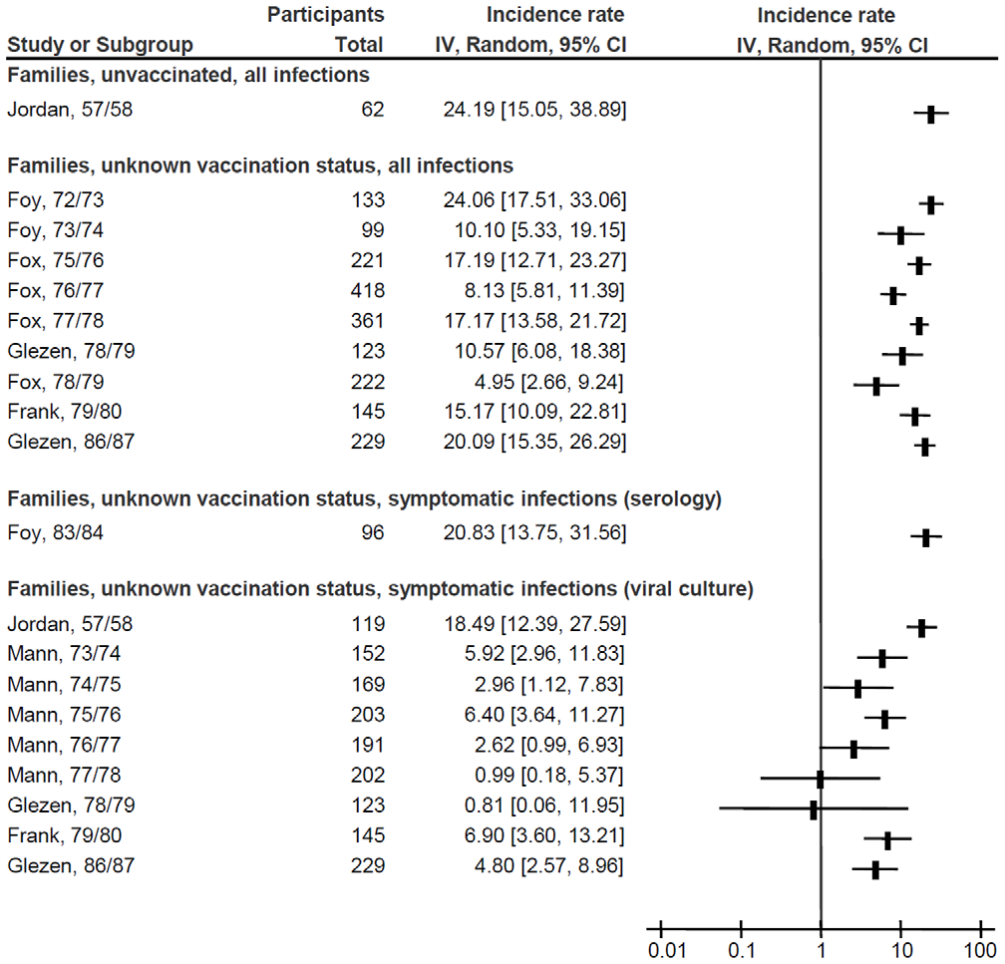
Forest plot of influenza incidence rates in adults sharing their households with children. Squares and horizontal lines through the squares represent incidence rates with 95% confidence intervals. Abbreviations: IV, inverse variance; CI, confidence interval; PCR, polymerase chain reaction.

Figures 1, 2 and 3 depict the influenza incidence rates in HCWs, adults working in non-healthcare settings and adults living in households with children, respectively. Pooled incidence rates of all subgroups, according to subpopulation, vaccination status, and diagnostic methods are presented in Table 1. No differences in incidence could be detected based on predominant influenza subtype in the study, or a requirement for fever in the definition of influenza-like illness (data not shown). Rates and 95% CI for all influenza infections ranged from 1.2% (95% CI, 0.9% to 1.7%) per season in vaccinated working adults to 24.2% (95% CI, 15.1% to 38.9%) in unvaccinated adults with exposure to children, whereas those for symptomatic infections ranged from 0.4% (95% CI, 0.1% to 1.6%) in vaccinated HCWs diagnosed by viral culture only to 20.8% (95% CI, 13.8% to 31.6%) in adults with exposure to children and unknown vaccination status, diagnosed by acute/convalescent or pre-/post-season serology. There was considerable heterogeneity within the subgroups: the only subgroup without significant heterogeneity was that of all infections in vaccinated working age adults, which included only two studies (I^2^ = 16%, *P* = 0.28).

**Table 1:** Influenza incidence rates according to subpopulation, diagnostic methods and vaccination status.

Subpopulation	Diagnostic methods	Vaccination status	Number of seasons	Number of Subjects	I^2^ (*P* value)	Incidence rate [95% confidence interval](n/100 population/season)
Families^1^	All infections	Unvaccinated	1	62	NA	24.19 [15.05, 38.89]
		Unknown	9	1,951	81% (<0.001)	13.56 [10.25, 17.94]
	Symptomatic infections (serology)	Unknown	1	96	NA	20.83 [13.75, 31.56]
	Symptomatic infections (culture)	Unknown	9	1,533	79% (<0.001)	4.96 [2.82, 8.71]
Working adults	All infections	Unvaccinated	4	4,373	93% (<0.001)	5.44 [3.01, 9.84]
		Vaccinated	2	3,717	16% (0.28)	1.20 [0.86, 1.68]
		Unknown	6	2,479	91% (<0.001)	9.13 [5.95, 14.01]
	Symptomatic infections (serology)	Unvaccinated	4	1,234	66% (0.03)	5.12 [3.08, 8.52]
		Vaccinated	3	2,619	68% (0.04)	3.04 [1.79, 5.15]
		Unknown	2	1,446	63% (0.10)	1.22 [0.48, 3.05]
	Symptomatic infections (culture)	Unvaccinated	8	9,393	90% (<0.001)	2.91 [1.78, 4.75]
		Vaccinated	8	14,017	89% (<0.001)	1.35 [0.76, 2.41]
	Symptomatic infections (PCR)	Unvaccinated	1	325	NA	10.77 [7.76, 14.94]
		Vaccinated	1	1,627	NA	5.16 [4.17, 6.40]
HCWs	All infections	Unvaccinated	10	2,273	66% (0.002)	18.69 [15.80, 22.11]
		Vaccinated	8	3.026	73% (<0.001)	6.49 [4.63, 9.09]
		Unknown	1	250	NA	11.20 [7.76, 16.17]
	Symptomatic infections (serology)	Unvaccinated	7	1,857	81% (<0.001)	7.54 [4.86, 11.70]
		Vaccinated	7	3,092	76% (<0.001)	4.81 [3.23, 7.16]
		Unknown	1	250	NA	8.00 [5.12, 12.49]
	Symptomatic infections (culture)	Unvaccinated	2	1,505	99% (<0.001)	6.24 [0.83, 46.88]
		Vaccinated	1	698	NA	0.43 [0.11, 1.61]
	Symptomatic infections (PCR)	Unknown	1	422	NA	2.37 [1.22, 4.61]

Abbreviations: CI, confidence interval; NA, not applicable; HCWs, healthcare workers.

1 Working-age adults living in households with children.

### Incidence rate of influenza in HCWs and healthy, working adults

Unvaccinated and vaccinated HCWs were compared to healthy, working adults with the same vaccination status by subgroup meta-analyses for all infections and symptomatic infections, respectively, using serology for influenza diagnosis (Table 2). There were too few data to compare studies of other subgroups. In all four subgroups, incidence rates in HCWs were higher than in working adults. Incidence rate ratios were higher for all infections than for symptomatic infections. Rates of all infections were found to be lower in vaccinated HCWs than in those who were unvaccinated.

**Table 2:** Incidence rates and incidence rate ratios of influenza infection of healthcare workers and non-healthcare workers from subgroup meta-analyses, according to vaccination status and diagnostic methods.

Vaccination status	Diagnostic methods	Subpopulation	Number of seasons	Number of subjects	Incidence rate [95% confidence interval](n/100 population/season)	Incidence Rate Ratio[95% confidence interval]
Unvaccinated	All infections	HCWs	10	2,273	18.69 [15.80, 22.11]	3.43 [1.20, 5.67]
		Working adults	4	4,373	5.44 [3.01, 9.84]	
Vaccinated	All infections	HCWs	8	3,026	6.49 [4.63, 9.09]	5.41 [2.79, 8.03]
		Working adults	2	3,717	1.20 [0.86, 1.68]	
Unvaccinated	Symptomatic infections (serology)	HCWs	7	1,857	7.54 [4.86, 11.70]	1.47 [0.44, 2.50]
		Working adults	4	1,234	5.12 [3.08, 8.52]	
Vaccinated	Symptomatic infections (serology)	HCWs	7	3,092	4.81 [3.23, 7.16]	1.58 [0.49, 2.67]
		Working adults	3	2,619	3.04 [1.79, 5.15]	

Abbreviations: HCWs, healthcare workers.

## Discussion

Our systematic review and meta-analyses of influenza incidence in HCWs and other healthy adults suggests that HCWs are at higher risk for influenza infection as compared to healthy adults working in non-healthcare settings. We found that the incidence rates of unvaccinated as well as vaccinated individuals with serologically proven, combined symptomatic and asymptomatic influenza is higher among HCWs than non-HCWs. However, we could not find a difference in the incidence of symptomatic influenza infection between HCWs and other working adults, although there was a trend to higher infection rates in HCWs, and the confidence limits do not exclude a 2.5 fold increase in risk. One potential explanation of the finding that HCWs are at higher risk of asymptomatic but not symptomatic influenza infection might be that their cumulative exposure to influenza (or influenza vaccine) over time is higher than that of other workers, so that prior immunity reduces symptom severity.

Our results also suggest that, among the populations studied, HCWs have similar risk to working age adults living in households with children, and that familial exposure to children substantially increases the risk of infection among non-HCWs, although, in accordance with our data analysis strategy, direct comparisons could not be performed and thus, these results should be interpreted with caution. It is well-known from family studies that influenza incidence is higher in children than in adults [21], [22], [24], [26], and observational studies have shown that living with children increases the risk for laboratory-confirmed influenza infection [9], [42]. Our results confirm that familial exposure to children should be taken into account when assessing influenza risk in routine clinical practice and controlled for in research studies. They also suggest that one mechanism of reducing the risk of failing to meet end-points in influenza vaccine efficacy trials in adults may be to selectively recruit adults with known familial exposure to children [25].

We were unable to locate any other systematic review or meta-analyses of influenza incidence in adults. Only one prospective observational study has directly compared influenza rates in HCWs and non-HCWs [9]. This study compared the incidence of influenza, as measured by influenza-like-illness and pre- and post-season serology among hospital healthcare workers and other working adults during the 2006/7 influenza season in Berlin, Germany. No difference in either symptomatic or asymptomatic influenza infection was identified in this cohort, although HCWs had an increased risk of acute respiratory infection (OR = 3.0, *P* = 0.04), and were more likely to have a pre-season antibody titer of ≥40 to influenza A/H3N2, suggesting greater exposure in prior seasons. One possibility for the differences between our study and that of Williams *et al.* is unmeasured differences in exposures to influenza, either in HCWs or other working adults. Although Williams *et al.* asked about overall person-contacts at work, work contacts with children may be particularly important (e.g. working in a daycare center) in working adults. Similarly, all hospital HCWs may not have the same risk of infection, and different types of HCWs may have been included in different studies.

Our study is important in that quantification of influenza risk in HCWs, particularly those working in acute care, is needed to support decisions regarding priorities for influenza vaccination and antiviral treatment or prophylaxis during pandemics. In addition, understanding influenza incidence in HCWs is important for implementation of infection control measures to reduce influenza transmission in hospitals. To date, four randomized controlled trials have now shown that vaccination of HCWs in long-term care is associated with a substantial decrease in mortality among residents [43]–[46]. If HCWs are indeed at particularly high risk for asymptomatic influenza infection, the greatest risk to frail elderly patients in hospitals may be from workers who are asymptomatically infected; vaccination then becomes the only strategy that will confer patient protection. The results of our study thus provide another strong argument for supporting universal influenza vaccination of hospital HCWs.

We anticipated heterogeneity between the studies due to variability in influenza rates from year to year, due to differences in predominating influenza strains from year to year, as well as to differences in study design. Therefore, we stratified our analyses according to exposure to children, vaccination status and diagnostic methods used, and pooled the weighted estimates of each study in these subgroups only. Incidence rates across different influenza subtypes were pooled as no difference between incidences in studies with different predominant subtypes could be detected. Nevertheless, there was substantial heterogeneity within subgroups. We suggest that this heterogeneity is partly related to study methodology (e.g. the variability in symptom complexes that resulted in specimen collection in symptomatic individuals), cross-reactive immunity between the previous vaccine strains, previous circulating strains and the new circulating strains and to the match of the influenza vaccine to the circulating strains in vaccinated individuals. Due to the complex cross-reactive immunity with previous circulating and vaccine strains, and the wide confidence limits on the proportion of infections due to any one subtype in individual studies, we did not attempt to further stratify for match of vaccine in those vaccinated.

Strengths of our study include the careful literature search and data collection, and *a priori* decision to perform subgroup meta-analyses only in the face of known clinical heterogeneity. This study also has limitations. First, we limited our search to two main databases and to the English literature for logistical reasons. Influenza incidence is often not the primary outcome of interest in these studies, necessitating a broad search strategy and wide screening. Nevertheless, we believe that we have sufficiently covered the existing literature for this study question. Second, newer diagnostic techniques may result in different findings for the detection of symptomatic influenza infection; given their greater sensitivity to lower viral loads (as may be seen in mild infection), it is possible that findings of studies using PCR detection in mild acute respiratory illness to define symptomatic infection will be different that those using culture and influenza-like-illness as a definition. Third, the existing tool for assessment of risk of bias of observational studies, the Newcastle Ottawa Scale [12], was not completely applicable. The tool that we thus needed to develop is not validated [11]. Fourth, even though we attempted to address potential confounding originating from pooling incidence rates by performing stratified analyses, some potential confounders could not be accounted for. As an example, we were unable to stratify for influenza seasons, taking into account variations of influenza rates over time. Nevertheless, we believe that our statistical analysis is robust enough to detect a difference originating from HCW status alone. Finally, as with all indirect meta-analyses, the comparisons in this study are indirect: data were obtained from different arms of various eligible studies, and the pooled incidence rates may not be directly comparable between groups. Limitations applicable to all indirect meta-analyses are relevant to our results as well and caution is warranted in interpretation.

In conclusion, our systematic review and meta-analysis provides valuable insights in the dynamics of influenza infection in adults. Our data suggest that HCWs are at higher risk of acquiring influenza infection as compared to adults working in non-healthcare settings, and that the rate of asymptomatic infections in particular might be considerably higher in HCWs. Adequately powered, prospective cohort studies that directly compare influenza rates in HCWs to those of working adults in non-healthcare settings are needed to confirm our findings. Additionally, future research should focus on the identification of high-risk subgroups among HCWs and other working adults.

## Supporting Information

Data S1 **Search strategy.**
(DOC)

Data S2 **List of excluded studies.**
(DOC)

Table S1 **Assessment of quality of included studies.**
(DOC)

Table S2 **Baseline characteristics of studies of rates of influenza in community-dwelling, working age adults assessing all infections or symptomatic influenza infection only.**
(DOC)

Table S3 **Baseline characteristics of studies of rates of influenza infection in healthcare workers assessing all infections or symptomatic influenza only.**
(DOC)

Table S4 **Risk of bias of included studies.**
(DOC)
