# Supplementary material for: Incidence of Influenza in Healthy Adults and Healthcare Workers: A Systematic Review and Meta-Analysis
Source: PLoS One. 2011 Oct 18;6(10):e26239. doi: 10.1371/journal.pone.0026239 (PMC3196543; doi:10.1371/journal.pone.0026239)
Supplement: Data S1 — Search strategy. (DOC) [file pone.0026239.s001.doc]

**Supplemental Data S1.** Search strategy

The following search strategy was used for OVID MEDLINE:

LIMIT (("clinical trial, all" or clinical trial).pt. or clinical trials as topic/ or clinical trial, phase i.pt. or clinical trials, phase i as topic/ or clinical trial, phase ii.pt. or clinical trials, phase ii as topic/ or clinical trial, phase iii.pt. or clinical trials, phase iii as topic/ or clinical trial, phase iv.pt. or clinical trials, phase iv as topic/ or controlled clinical trial.pt. or controlled clinical trials as topic/ or multicenter study.pt. or multicenter studies as topic/ or randomized controlled trial.pt. or randomized controlled trials as topic/ or cohort studies/ or longitudinal studies/ or follow-up studies/ or prospective studies/ or case-control studies/ or retrospective studies/ or cross-sectional studies/ or morbidity/ or incidence/ or prevalence/ or mortality/ or cause of death/ or fatal outcome/ or hospital mortality/ or infant mortality/ or maternal mortality/ or survival rate/ or survival analysis/ or disease-free survival/ or population surveillance/ or sentinel surveillance/ or Disease Outbreaks) AND (limit (influenza, Human/ or exp influenzavirus a/ or exp influenzavirus b/ or exp influenzavirus c/ or influenza Vaccines/ or (flu or flus*).mp.) TO (English language and humans))) NOT (("all child (0 to 18 years)" or "all aged (65 and over)")

The following search strategy was used for EMBASE:

LIMIT ((clinical trial/ or controlled clinical trial/ or phase 1 clinical trial/ or phase 2 clinical trial/ or phase 3 clinical trial/ or phase 4 clinical trial/ or controlled study/ or randomized controlled trial/ or crossover procedure/ or double blind procedure/ or drug screening/ or intervention study/ or clinical study/ or multicenter study/ or open study/ or prevention study/ or therapy/ or "trial of labor"/ or triple blind procedure/ or cohort analysis/ or prospective study/ or morbidity/ or incidence/ or prevalence/ or seroprevalence/ or health survey/ or epidemic/ or exp "cost benefit analysis"/ or follow up/ or longitudinal study) and (influenza vaccine/ or influenza virus a h1n1/ or influenza virus a h2n2/ or influenza virus a h3n2/ or influenza virus a h3n8/ or influenza virus a h5n1/ or influenza virus a h5n2/ or influenza virus a h7n7/ or influenza virus a h9n2/ or hong kong influenza/ or influenza virus/ or influenza/ or influenza virus a/ or swine influenza virus/ or avian influenza/ or influenza virus b/ or influenza virus c/ or influenza vaccination)) TO (human and English language).
