# Supplementary material for: Incidence of Influenza in Healthy Adults and Healthcare Workers: A Systematic Review and Meta-Analysis
Source: PLoS One. 2011 Oct 18;6(10):e26239. doi: 10.1371/journal.pone.0026239 (PMC3196543; doi:10.1371/journal.pone.0026239)
Supplement: Data S2 — List of excluded studies. (DOC) [file pone.0026239.s002.doc]

**Supplemental Data S2.** List of excluded studies

- *Duplicates of included publications:*

Madore HP *et al.* J Clin Microbiol 1983;18(6):1345-50

Keitel WA *et al*. Am J Epidemiol 1988;127(2):353-64

Sullivan KM *et al*. J Public Health 1993;83:1712-6

Epstein SL. J Infect Dis 2006;193:49–53

- *Studies not covering the entire influenza season:*

Oker-Blom N *et al.* BMJ 1970;3:676-8

Smorodintsev AA *et al.* Bull World Health Organ 1970;42(6):865-72

Nafta I *et al.* Bull World Health Organ 1970;42(3):423-7

Quarles JM *et al.* Antiviral Res 1981;1(3):149-55

Dolin R *et al.* N Engl J Med 1982;307:580-4

Reuman PD *et al.* Antiviral Res 1989;11(1):27-40

Hayden FG *et al*. N Engl J Med 1999;341:1336-43

Monto AS *et al.* JAMA 1999;282(1):31-35

- *Studies focusing on specific influenza subtypes only:*

Muldoon RL *et al.* Am Rev Respir Dis 1976;113(4):487-91

Delem A *et al*. J Hyg 1979;83(2):221-9

Pyhälä R. J Hyg 1980;84(2):237-45

Monto AS *et al*. J Infect Dis 1982;145(1):57-64

Frank AL and Taber LH. J Med Virol 1983;12(1):17-23

Frank AL *et al.* Am J Epidemiol 1987;125(4):576-86

Brady MT *et al.* Antimicrob Agents Chemother 1990;34(9):1633-6

Powers DC *et al.* J Infect Dis 1995;171(6):1595-9

- *Studies reporting influenza outbreaks:*

Blumenfeld HL *et al.* J Clin Invest 1959;38(1 Part 2):199-212

Arroyo JC *et al*. Am J Infect Control 1984;12(6):329-34

Nicholls S *et al.* Commun Dis Public Health 2004;7(4):272-7

Guy R *et al.* Aust N Z J Public Health 2005;29:540-3

Apisarnthanarak A *et al*. Infect Control Hosp Epidemiol 2008;29(8):777-80

- *Studies including children only or all age groups (with >2% children or proportion of children unknown), not allowing to separate children from adults:*

Brown P *et al.* Am J Epidemiol 1969;90(4):336-42

Philip RN *et al.* Am J Epidemiol 1969;90(6):471-83

Gill PW and Murphy AM. Med J Aust 1977;2:761-5

Jennings LC *et al.* J Hyg 1978;81(1):49-66

Monto AS *et al.* J Infect Dis 1970;122(1):16-25

Ochiai H *et al.* Microbiol Immunol 1986;30:1151-65

Gill PW *et al.* Med J Aust 1991;155:362-7

Edwards KM *et al.* Infect Dis 1994;169(1):68-76

- *Studies including university or college students, military personnel or prisoners:*

Hayslett J *et al.* Am Rev Respir Dis 1962;85:1-8

Wendel HA *et al.* Clin Pharmacol Ther 1966;7(1):38-43

Dawkins AT *et al.* JAMA 1968;203:1095-9

Peckinpaugh RO *et al.* Bull World Health Organ 1969;41(3):404-6

Stark JE *et al.* Thorax 1970;25:649-55

Mogabgab WJ and Leiderman E. JAMA 1970;211(10):1672-6

Bruj J et Farník J. Zentralbl Bakteriol Orig 1970;213(1):28-35

Mate J *et al.* Acta Microbiol Acad Sci Hung 1970;17(3):285-96

Leibovitz A *et al.* J Infect Dis 1971;124(5):481-7

Evans AS *et al.* Am Rev Respir Dis 1973;108:1311-9

Stiver HG *et al.* N Engl J Med 1973;289:1267-71

Ruben FL *et al.* Arch Intern Med 1973;132:568-571

André FE *et al.* Postgrad Med J 1976;52;352-9

Morris CA *et al.* Dev Biol Stand 1976;33:197-201

Dodge JS. NZ Med J 1978;87:79-82

Leigh Hammond M *et al.* Med J Aust 1978;1:301-3

Rocchi G *et al.* Hyg 1979;83(3):383-90

Monto AS *et al.* JAMA 1979;241:1003-7

Pettersson RF *et al.* J Infect Dis 1980;142(3):377-83

Foy HM *et al.* JAMA 1981;245:1736-40

Lebiush M *et al.* Military Medicine 1982;147:43-8

Sonoguchi T *et al.* J Infect Dis 1986;153(1):33-40

Aoki FY *et al.* Military Medicine 1986;151(9):459-65

Wale MCJ. J Roy Nav Med Serv 1989;75:13-8

Gray CG *et al.* Military Medicine 2001;166(9):759-63

Strickler JK *et al.* Emerg Infect Dis 2007;13(4):617-9

- *Studies in other communities with crowded living conditions:*

El Bashir H *et al.* Emerg Infect Dis 2004;10(10):1882-3

- *Studies in high-risk populations (including >50% of subjects being >65 years of age, or proportion of elderly subjects not reported):*

Petrilli FL *et al.* Boll Ist Sieroter Milan 1975;54(4):323-30

De Barbieri A *et al.* Dev Biol Stand 1976;33:213-9

Chotkowski LA *et al.* Conn Med. 1981 Dec;45(12):773

Cruijff M *et al.* Vaccine 1999;17:426-32

Belongia EA *et al.* J Infect Dis 2009;199(2):159-67

- *Studies not systematically including laboratory confirmation of influenza infection:*

Solov’ev VD. Bull World Health Organ 1969;41(3):683-8

Waldman RH *et al.* Bull World Health Organ 1969;41(3):543-8

Davies JE *et al.* Br J Clin Pract. 1972;26(10):469-71

Davies JE *et al.* Br J Clin Pract. 1973;27(6):219-21

Robert JA *et al.* Br J Sports Med 1988;22(4):161-2

Grotto I *et al.* Clin Infect Dis 1998;26(4):913-7.

Belshe RB *et al.* Clin Infect Dis 2004;39(7):920-7

Kramer JS *et al.* Am J Health Syst Pharm. 2006;63(21):2111-5

- *Studies using rapid antigen tests predominantly (>50% of cases) for influenza diagnosis:*

Kawai N *et al.* Vaccine 2003;21(31):4507-13

Ito Y *et al.* J Infect Chemother 2006;12:70–2

Kawana A *et al.* Jpn J Infect Dis 2006;59(6):377-9

Yamada H *et al.* Jpn J Clin Pharmacol Ther 2007;38:323-9

- *Studies reporting influenza seroprevalence rather than incidence:*

Hosny AH *et al.* J Egypt Public Health Assoc 1966;41(5):281-7

Zimmer SM *et al.* PLoS ONE 5(7): e11601

Tan DS and Omar M. Med J Malaysia 1974;29(1):17-23

- *Case-studies:*

Gill PW *et al.* Med J Aust 1971;1(19):1005-8

Kasel JA *et al.* Proc Soc Exp Biol Med 1979;161(4):519-21

Shimokata K *et al.* Jpn J Med 1988;27(3):286-90

Bellei N *et al.* Respirology 2007;12(1):100-3

- *Reviews:*

Jackson GG and Stanley ED. JAMA 1976;235(25):2739-42

- *Studies not reporting annual figures:*

Evans AS *et al.* Am J Epidemiol 1971;93(6):463-71

Monto AS and Cavallaro JJ. Am J Epidemiol 1971;94(3):280-9

Monto AS and Kioumehr F. Am J Epidemiol 1975;102(6):553-63

Monto AS *et al*. Am Rev Respir Dis 1975;111:27-36

Delem A and Jovanovic D. J Infect Dis 1978;137(2):194-6

Foy HM *et al.* J Infect Dis 1981;143(5):700-6

Hope-Simpson RE. J Hyg 1984;92(3):303-6

Frank AL *et al.* J Infect Dis 1985;151(1):73-80

Monto AS *et al.* Am J Epidemiol 1985;121(6):811-22

Monto AS *et al.* Am J Epidemiol 1986;124(3):359-67
