# Supplementary material for: Incidence of Influenza in Healthy Adults and Healthcare Workers: A Systematic Review and Meta-Analysis
Source: PLoS One. 2011 Oct 18;6(10):e26239. doi: 10.1371/journal.pone.0026239 (PMC3196543; doi:10.1371/journal.pone.0026239)
Supplement: Table S1 — Assessment of quality of included studies. (DOC) [file pone.0026239.s003.doc]

**Table S1.** Assessment of quality of included studies

| **Bias** | **Minimal** | **Low** | **Moderate** | **High** | **Unclear** |
| --- | --- | --- | --- | --- | --- |
| **Selection^1^** | - Consecutive unselected population - Sample selected from general population rather than a select group - Follow up or assessment time explained | - Sample selected from large population but selection criteria not defined - A select group of population (based on race, ethnicity, residence etc.) studied | - Sample selection ambiguous but sample may be representative - Eligibility criteria not explained - Follow up or assessment time not explained | - Sample selection ambiguous and sample likely not representative - A very select population studied making it difficult to generalize findings | - Not reported or unclear |
| **Outcome assessment (symptomatic infections only)** | - Active follow-up, testing for any symptoms of influenza | - Active follow-up, testing only if >1 symptom - Testing based on self-reported illness, any symptoms of influenza | - Active follow-up, fever >38.0°C required for testing - Testing based on self-reported illness, but only if >1 symptom of influenza | - Testing based on self-reported illness, fever >38.0°C required for testing | - Not reported or unclear |
| **Attrition** | - 0-10% attrition and reasons for loss of follow up explained - All subjects from initiation of study to the final outcome assessment were accounted for | - 0-10% attrition and reasons for loss of follow up not explained - 11-20% attrition, reasons for loss of follow up explained | - 11-20% attrition but reasons for loss of follow up not explained - >20% attrition but reasons for loss of follow up explained - Not all subjects from initiation of study to final outcome assessment were accounted for | - >20% attrition, reasons for loss of follow up not explained | - Not reported or unclear |

^1^ For studies in health care workers, ‘unselected population’ equates to any subjects working in a health care facility.
