# Supplementary material for: Incidence of Influenza in Healthy Adults and Healthcare Workers: A Systematic Review and Meta-Analysis
Source: PLoS One. 2011 Oct 18;6(10):e26239. doi: 10.1371/journal.pone.0026239 (PMC3196543; doi:10.1371/journal.pone.0026239)
Supplement: Table S2 — Baseline characteristics of studies of rates of influenza in community-dwelling, working age adults assessing all infections or symptomatic influenza infection only. (DOC) [file pone.0026239.s004.doc]

**Table S2** Baseline characteristics of studies of rates of influenza in community-dwelling, working age adults assessing all infections or symptomatic influenza infection only

| **Source** | **Study design**  **Population**  **Country of origin** | **Diagnostic methods** | **Influenza seasons** | **Circulating influenza subtypes^1^** | **Number of subjects included in meta-analysis** |
| --- | --- | --- | --- | --- | --- |
| Jordan *et al*., 1958 [26] | Prospective cohort study  Families with children  USA | All infections: Serology  Symptomatic infections: Viral culture for respiratory illness | 1957-8 | H3N2 | All infections: 62 (unvaccinated)  Symptomatic infections: 119 (all subjects) |
| Mair *et al.*, 1974 [30] | Randomized controlled trials of vaccine Community  United Kingdom | All infections: Serology  Symptomatic infections: Serology and viral culture for reported influenza-like illness | 1972-3 | H3N2 | All infections: 151 (unvaccinated)  Symptomatic infections: 169 (vaccine), 296 (placebo) |
| Foy *et al.*, 1976 [22] | Prospective cohort study  Families with children  USA | All infections: Serology | 1972-3  1973-4 | H3N2, B  B, H3N2 | 133  99 |
| Mann *et al.*, 1981 [31] | Prospective cohort study  Families with children  England and Wales | Symptomatic infections: Viral culture or acute/convalescent serology for reported acute respiratory or febrile illness | 1973-4  1974-5  1975-6  1976-7  1977-8 | H3N2  H3N2  H3N2, B  H3N2  H3N2, H1N1 | 152  169  203  191  202 |
| Fox *et al.*, 1982 [21] | Prospective cohort study  Families with children  USA | All infections: Serology and viral culture | 1975-6  1976-7  1977-8  1978-9 | H3N2, B  H3N2, B  H3N2, H1N1, B B, H1N1, H3N2 | 221  418  361  222 |
| Frank *et al.*, 1983 [5] | Prospective cohort study  Families with children  USA | All infections: Serology and viral culture  Symptomatic infections: Viral culture for reported acute respiratory illness | 1979-80 | B | 145 |
| Monto and Sullivan*,* 1993 [33] | Prospective cohort study  Community  USA | All infections: Serology and viral culture | 1976-7  1977-8  1978-9  1979-80  1980-1 | B  H3N2, H1N1  H1N1  B  H3N2, H1N1 | 467  391  376  419  414 |
| Glezen *et al.,* 1991 [24] | Prospective cohort study  Families with children  USA | All infections: Serology and viral culture  Symptomatic infections: Viral culture for reported acute respiratory illness | 1978-9  1986-7 | H1N1  H1N1 | 123  229 |
| Foy *et al.*, 1987 [23] | Randomized controlled trial of interferon  Families with children  USA | Symptomatic infections: Viral culture and acute/convalescent serology for reported acute respiratory illness | 1983-4 | B | 96 |
| Tannock *et al.*, 1988 [37] | Randomized controlled trial of interferon Community  Australia | All infections: Serology and viral culture | 1984 | H3N2 | 412 |
| De Wolf *et al.*, 1988 [18] | Prospective cohort study  MSM  The Netherlands | Symptomatic infections: Acute/convalescent serology for reported acute febrile illness | 1984-5  1985-6 | H3N2  B, H3N2 | 723  723 |
| Tannock *et al.*, 1993 [38] | Prospective cohort study  Community  Australia | All infections: Serology and viral culture  Symptomatic infections: Acute/convalescent serology and viral culture for reported influenza-like symptoms | 1987 | B, H1N1, H3N2 | 454 |
| Bridges *et al.,* 2000 [17] | Randomized controlled trial of vaccine  Working adults  USA | Symptomatic infections: Viral culture for influenza-like illness | 1997-8  1998-9 | H3N2  H3N2, B | 138 (vaccine)  137 (placebo)  141 (vaccine)  137 (placebo) |
| Treanor *et al.*, 2007 [39] | Randomized controlled trial of vaccine  Community  USA | Symptomatic infections: Viral culture for acute respiratory tract symptoms or fever | 2004-5 | H3N2, B | 301 (vaccine)  153 (placebo) |
| Ohmit *et al.*, 2006 [34] | Randomized controlled trial of vaccine  Community and university sites  USA | Symptomatic infections: Serology and viral culture for at least two respiratory  or systemic signs or symptoms | 2004-5 | H3N2, B | 730 (vaccine)  146 (placebo) |
| Ohmit *et al.,* 2008 [35] | Randomized controlled trial of vaccine  Community and university sites  USA | Symptomatic infections: Serology and viral culture/PCR for at least two respiratory or systemic signs or symptoms | 2005-6 | H3N2, B | 1,720 (vaccine)  338 (placebo) |
| Beran *et al.*, 2009 [16] | Randomized controlled trial of vaccine  Community  Czech Republic | Symptomatic infections: Viral culture for influenza-like illness | 2005-6 | B, H1N1, H3N2 | 4,137 (vaccine)  2,066 (placebo) |
| Jackson *et al.*, 2010 [25] | Randomized controlled trial of vaccine  Community  USA | All infections: Serology and viral culture  Symptomatic infections: Viral culture for influenza-like illness | 2005-6  2006-7 | H3N2, B  H3N2, H1N1, B | 1,706 (vaccine)  1,725 (placebo)  2,011 (vaccine)  2,043 (placebo) |
| Beran *et al.*, 2009 [15] | Randomized controlled trial of vaccine  Community  Czech Republic and Finland | Symptomatic infections: Viral culture with influenza-like illness | 2006-7 | H3N2, H1N1, B | 5,103 (vaccine)  2,549 (placebo) |
| Monto *et al.*, 2009 [32] | Randomized controlled trial of vaccine  Community  USA | Symptomatic infections: PCR and viral culture for at least two respiratory  or systemic signs or symptoms | 2007-8 | H3N2, B, H1N1 | 1,627 (vaccine)  325 (placebo) |

Abbreviations: MSM, men who have sex with men;

^1^underlined subtypes account for >75% of subtypes in study population
