# Supplementary material for: Incidence of Influenza in Healthy Adults and Healthcare Workers: A Systematic Review and Meta-Analysis
Source: PLoS One. 2011 Oct 18;6(10):e26239. doi: 10.1371/journal.pone.0026239 (PMC3196543; doi:10.1371/journal.pone.0026239)
Supplement: Table S3 — Baseline characteristics of studies of rates of influenza infection in healthcare workers assessing all infections or symptomatic influenza only. (DOC) [file pone.0026239.s005.doc]

**Table S3** Baseline characteristics of studies of rates of influenza infection in healthcare workers assessing all infections or symptomatic influenza only

| **Source** | **Setting**  **Population**  **Country of origin** | **Diagnostic methods** | **Influenza seasons** | **Circulating Influenza subtypes^1^** | **Number of subjects included in meta-analysis** |
| --- | --- | --- | --- | --- | --- |
| Waldman *et al.*, 1969 [40] | Randomized controlled trial of vaccine University and hospital employees  USA | Symptomatic infections: Acute/convalescent serology for reported illness resembling influenza | 1967-8 | H2N2 | 81 (vaccine)  25 (placebo) |
| Feery *et al.*, 1979 [20] | Prospective studies of vaccine  Hospital and laboratory employees  Australia | Symptomatic infections: Viral culture for acute respiratory illness | 1976 | H3N2 | 698 (HCWs, vaccinated)  1,304 (HCWs, unvaccinated)  480 (lab, vaccinated)  583 (lab, unvaccinated) |
| Keitel *et al.,* 1997 [27] | Prospective cohort study  Healthcare workers and other healthy adults  USA | All infections: Serology and viral culture  Symptomatic infections: Serology and viral culture for reported acute respiratory or influenza-like illness | 1983-4  1984-5  1985-6  1986-7  1987-8 | H1N1, B  H3N2  B  H1N1  H3N2, B | 298 (unvaccinated)  300 (vaccinated)  241 (unvaccinated)  457 (vaccinated)  253 (unvaccinated)  577 (vaccinated)  217 (unvaccinated)  723 (vaccinated)  145 (unvaccinated)  789 (vaccinated) |
| Kumpulainen *et al*., 1997 [28] | Prospective cohort study  Municipal homemakers  Finland | Symptomatic infections: Acute/convalescent serology and antigen test for reported acute respiratory illness | 1990-1 | B, A | 165 (vaccinated)  678 (unvaccinated) |
| Wilde *et al.*, 1999 [41] | Randomized controlled trial of vaccine Hospital-based healthcare workers  USA | All infections: Serology | 1992-3  1993-4  1994-5 | H3N2, B  H3N2  B, H3N2 | 52 (vaccine)  50 (placebo)  51 (vaccine)  52 (placebo)  77 (vaccine)  77 (placebo) |
| Elder et al., 1996 [19] | Prospective cohort study  Healthcare workers in acute care  U.K. | All infections: Serology | 1993-4 | A, B | 518 |
| Sirivichayakul et al., 2000 [36] | Prospective cohort study  Nurse assistant students  Thailand | Symptomatic infections: Viral culture for reported influenza-like illness | 1998-9 | A, B | 201 |
| Williams *et al.*, 2010 [9] | Prospective cohort study  Healthcare workers in acute care Germany | All infections: Serology  Symptomatic infections: Serology for acute respiratory illness | 2006-7 | H3N2, H1N1, B | 250 |
| Loeb et al., 2009 [29] | Randomized controlled trial of masks Nurses in acute care  Canada | All infections: Serology and PCR  Symptomatic infections: PCR for reported symptoms of influenza | 2008-9 | H1N1, B, H3N2 | 212 (surgical mask)  210 (N95 respirator) |

Abbreviations: HCWs, healthcare workers.

^1^underlined subtypes account for >75% of subtypes in study population
