# Supplementary material for: Incidence of Influenza in Healthy Adults and Healthcare Workers: A Systematic Review and Meta-Analysis
Source: PLoS One. 2011 Oct 18;6(10):e26239. doi: 10.1371/journal.pone.0026239 (PMC3196543; doi:10.1371/journal.pone.0026239)
Supplement: Table S4 — Risk of bias of included studies. (DOC) [file pone.0026239.s006.doc]

**Table S4.** Risk of bias of included studies

| **Source** | **Selection** | **Outcome assessment** | **Attrition** | **Total risk of bias** |
| --- | --- | --- | --- | --- |
| Jordan *et al*., 1958 | Moderate | Low | Minimal | Moderate |
| Mair *et al.*, 1974 | Low | Low | Low | Low |
| Foy *et al.*, 1976 | Low | n.a. | Low | Low |
| Mann *et al.*, 1981 | Low | Low | Minimal | Low |
| Fox et al., 1982 | Low | n.a. | Minimal | Low |
| Frank *et al.*, 1983 | Low | Minimal | Minimal | Low |
| Monto and Sullivan*,* 1993 | Low | n.a. | Minimal | Low |
| Glezen et al., 1991 | Low | Minimal | Minimal | Low |
| Foy *et al.*, 1987 | Low | Minimal | Low | Low |
| Tannock *et al.*, 1988 | Minimal | n.a. | Minimal | Minimal |
| De Wolf *et al.*, 1988 | High | High | Minimal | High |
| Tannock *et al.*, 1993 | Minimal | Low | Minimal | Low |
| Bridges *et al.,* 2000 | Moderate | Moderate | Minimal | Moderate |
| Treanor *et al.*, 2007 | Minimal | Minimal | Minimal | Minimal |
| Ohmit *et al.*, 2006 | Minimal | Moderate | Minimal | Moderate |
| Ohmit *et al.,* 2008 | Minimal | Moderate | Minimal | Moderate |
| Beran *et al.*, BMC ID 2009 | Minimal | Low | Minimal | Low |
| Jackson *et al.*, 2010 | Minimal | Low | Minimal | Low |
| Beran *et al.*, JID 2009 | Minimal | Low | Minimal | Low |
| Monto *et al.*, 2009 | Minimal | Moderate | Minimal | Moderate |
| Waldman *et al.*, 1969 | Low | Low | Minimal | Low |
| Feery *et al.*, 1979 | Low | Low | Minimal | Low |
| Keitel *et al.,* 1997 | Low | Low | Moderate | Moderate |
| Kumpulainen *et al*., 1997 | Low | Low | High | High |
| Wilde *et al.*, 1999 | Low | n.a. | Unclear | Low |
| Elder et al., 1996 | Low | n.a. | Minimal | Low |
| Sirivichayakul et al., 2000 | Low | Moderate | Minimal | Moderate |
| Williams *et al.*, 2010 | Low | Low | Moderate | Moderate |
| Loeb et al., 2009 | Low | Minimal | Minimal | Low |

Abbreviations: n.a., not applicable
